# A manual-based individual therapy to improve metacognition in schizophrenia: protocol of a multi-center RCT

**DOI:** 10.1186/1471-244X-14-27

**Published:** 2014-02-03

**Authors:** Rozanne JM Van Donkersgoed, Steven De Jong, Mark Van der Gaag, André Aleman, Paul H Lysaker, Lex Wunderink, GHM Pijnenborg

**Affiliations:** 1 Department of clinical psychology and experimental psychopathology, Faculty of Behavioral and Social Sciences, University of Groningen, Grote Kruisstraat 2/1, 9712 TS Groningen, the Netherlands; 2 Department of Education and Research, Friesland Mental Health Care Services, PO Box 932, 8901 BS Leeuwarden, the Netherlands; 3 Department of Psychotic Disorders, GGZ-Drenthe, Dennenweg 9, 9404 LA Assen, the Netherlands; 4 Parnassia Psychiatric Institute, Prinsegracht 63, 2512 EX The Hague, the Netherlands; 5 Department of Neuroscience, BCN Neuroimaging Center, University of Groningen, Medical Center Groningen, Antonius Deusinglaan 2, 9713 AW Groningen, the Netherlands; 6 Roudebush VA Medical Center, Indiana School of Medicine, Department of Psychiatry, 1481 West 10th Street, Indianapolis, IN 46292, USA; 7 Department of Psychiatry, University Medical Center Groningen, PO Box 30.001, 9700 RB Groningen, the Netherlands

**Keywords:** Schizophrenia, Psychosis, Metacognition, Treatment, Therapy, Functioning

## Abstract

**Background:**

Metacognitive dysfunction has been widely recognized as a feature of schizophrenia. As it is linked with deficits in several aspects of daily life functioning, improvement of metacognition may lead to improvement in functioning. Individual psychotherapy might be a useful form of treatment to improve metacognition in patients with schizophrenia; multiple case reports and a pilot study show promising results. The present study aims to measure the effectiveness of an individual, manual-based therapy (Metacognitive Reflection and Insight Therapy, MERIT) in improving metacognition in patients with schizophrenia. We also want to examine if improvement in metacognitive abilities is correlated with improvements in aspects of daily life functioning namely social functioning, experience of symptoms, quality of life, depression, work readiness, insight and experience of stigma.

**Methods/Design:**

MERIT is currently evaluated in a multicenter randomized controlled trial. Thirteen therapists in six mental health institutions in the Netherlands participate in this study. Patients are randomly assigned to either MERIT or the control condition: treatment as usual (TAU).

**Discussion:**

If proven effective, MERIT can be a useful addition to the care for schizophrenia patients. The design brings along some methodological difficulties, these issues are addressed in the discussion of this paper.

**Trial registration:**

Current Controlled Trials: ISRCTN16659871.

## Background

Metacognitive dysfunction has been widely recognized as a feature of schizophrenia [1,2] and is linked with deficits in daily life functioning [3-5]. Over the years, the construct of metacognition has been defined in different ways. All definitions describe a general capacity to think about thinking [6]. Flavell [7] was one of the first to use the term. In his view metacognition is closely related to ‘cognitive monitoring’: the ability to observe one’s own cognitive processes and to detect errors in these processes. Moritz & Woodward [8] provide a more specific definition that targets dysfunctional thought processes in particular, defining metacognition as ‘being aware of cognitive distortions’. Wells [9] includes thinking about feelings. He also describes the important role of metacognition in eliciting reactions triggered by these thoughts and feelings. Based on the work of Semerari et al. on metacognition [10], Lysaker et al. [11] propose to integrate different definitions and propose a definition that involves four fundamental aspects:

– Self-reflectivity: the ability to think about one’s own thoughts and emotions;

– Understanding the other’s mind: the ability to think about the thoughts and emotions of others;

– Decentration: the ability to understand that you are not the center of the world and people’s lives continue when you are not around;

– Mastery: One’s ability to use the three aspects above to define psychological problems and adequately deal with them.

In this definition, metacognition refers to a spectrum of activities, which involves thinking about thinking and stretches from consideration of discrete psychological phenomena (for example: recognizing an isolated thought) to the synthesis of discrete perceptions into an integrated representation of self and others [12]. Metacognitive capacities “allow persons to form a detailed picture of their own mental states, of the wishes and intentions of the others, and of the inner and social cues that trigger psychological pain, and thereby to cope with challenges and solve complex social problems […]. In the larger frame they make it possible for persons to make sense of their dilemmas, to find meaning in life, and to ultimately adapt to a changing environment” [2]. In this way metacognitive dysfunction is more than just a missing skill. It is the inability to make complex sense of experience and integrate interpersonal information into a larger whole [12].

Several instruments have been developed to assess elements of metacognition. Most of these instruments capture more discrete aspects of the concept as it is defined above, for example the Meta-Cognitions Questionnaire (MCQ) [13] which measures beliefs about worries and intrusive thoughts and the Davos Assessment of Cognitive Biases Scale (DACOBS) [14], measuring cognitive biases. Semerari et al. [10] developed the Metacognition Assessment Scale (MAS) based on the four elements of metacognition. This instrument was originally designed to measure metacognition in people with personality disorders. Lysaker et al. [11] have shortened this instrument and adapted it for use with people with psychotic disorders (MAS-A).

Metacognition is linked to aspects of daily life function of patients with schizophrenia in several ways. Better metacognitive mastery is linked to better social cognition and more insight [2,15] and metacognition has been found to mediate the impact of neurocognitive deficits on social function, after controlling for symptoms [16]. Lower levels of metacognition correlate with less favorable reports of the subjective experience of recovery [17] and lower levels of functional competence [18]. In addition to these daily life aspects, impaired metacognition has also been associated with low quality of therapeutic alliance [19] and more severe negative symptoms [11,20,21]. Moreover, lower levels of metacognition predict future severity of negative symptoms, even after controlling for concurrent levels of negative symptoms [22].

Based on the above, it is hypothesized that improved metacognition will result in improved daily life functioning. Metacognition might be a feasible target for treatment of people with psychotic disorders. If an intervention would be capable of improving metacognitive capacity, it would enable persons with schizophrenia to achieve more complex understanding of themselves and others and thus find ways to actively direct their recovery process. Individual psychotherapy might be a useful form of treatment to improve metacognition. Several forms of psychotherapy have successfully promoted metacognitive capacity in persons with various mental disorders other than psychosis [23], including work from a psychoanalytic [24] and a cognitive frame of reference [25]. It has also been suggested that some of these therapeutic procedures could be modified for treatment of people with schizophrenia [26]. If metacognition is conceptualized as a trait-like capability that varies along a continuum from good to impoverished, psychotherapy might provide a place to practice such capacities in increasing degrees of complexity, leading to enhancement of metacognitive capacity. Manualized procedures for a metacognitive psychotherapy for schizophrenia have been proposed [26] and multiple detailed case reports and a pilot study have documented the acceptability of the treatment and positive outcomes [27-33]. The present paper presents the design of a randomized controlled multicenter trial that aims to measure the effectiveness of the Metacognitive Reflection and Insight Therapy (MERIT) in improving metacognition.

### Research aims

Primary objective of this study is to investigate whether Metacognitive Reflection and Insight Therapy improves metacognitive abilities in people with schizophrenia. Secondary objective is to examine whether improvements in metacognitive capacity lead to improvements in quality of life, social functioning, depression, symptoms, stigma sensitivity and work readiness. Neurocognition is adopted in the assessment as well, as it may function as a mediator between metacognition and functioning. A cost-effectiveness analysis will be performed at the end of the study.

## Methods/Design

The study is funded by Fonds NutsOhra, GGZ Drenthe, GGZ Friesland and the Institute for Post master Psychology in the Northern Netherlands (PPO). The study has been approved by the medical ethical board of University Medical Center Groningen, Groningen (number: METc2013.124, date: november 2013), and is conducted in accordance with the principles of the Declaration of Helsinki. Trial number: ISRCTN16659871 (Current Controlled Trials).

### Design

The study is designed as a multicenter randomized controlled trial including an intervention group receiving Metacognitive Reflection and Insight Therapy (MERIT) and a control group receiving treatment as usual (TAU).

### Participants/setting

Participants in the study are people with schizophrenia or schizoaffective disorder and impaired metacognitive abilities. A total of 120 participants will be included from six mental health care institutions in the Netherlands. Thirteen therapists who work in these facilities will be trained to give the therapy.

Inclusion criteria are:

Impaired metacognitive skills, as measured by the Metacognition Assessment Scale (MAS-A);

Diagnosis of schizophrenia or schizoaffective disorder, according to DSM-IV-TR criteria;

Being able to give informed consent;

18 years or older;

No change in medication in the past thirty days.

Exclusion criteria are:

Acute psychosis (mean score of PANSS positive symptoms >4);

Co-morbid neurological disorder;

Substance dependence (not substance abuse as measured with the MINI Plus);

Impaired intellectual functioning (IQ <70)

### Sample size calculation

Sample size was computed with a two-sided test using the IBM SPSS Sample Power program (Biostat, M. Borenstein), http://www.power-analysis.com/about_biostat.htm. The effect size of MERIT is not known. We chose to adopt the common convention in such instances, and set our effect size at 0.5 [34]. This is based on the consideration that should the effect size be lower, results would probably not be clinically relevant [35,36]. In order to show medium effect sizes (0.5) with an alpha of 0.05 and a power of 0.80, a minimum of 48 subjects in both groups is required. This means that there is an 80% likelihood that the study will detect a statistically significant effect if such exists, and allows us to conclude that the mean metacognition score differs for MERIT versus TAU. Because this is a time consuming trial with people with a severe mental illness, we assume that the percentage of dropouts will be 20%. This means that for every 120 subjects enrolled in the study, 24 will be lost to follow-up or withdraw informed consent and will be excluded from the analysis. Therefore we will select 60 patients in the intervention group and 60 patients in the control group including 12 extra participants in each group to compensate for the anticipated missing data.

### Procedure

Thirteen therapists in six Dutch mental health care institutions will select all patients in their caseload that meet the inclusion criteria. They will answer four screening questions concerning these patients’ metacognitive abilities on a 10 point scale (see Section Screening for the screening questions). The therapists approach the patients that score below the cut-off of the screening and ask them if they are interested in participation. The names of the interested patients will be coded by the therapist. Out of these coded names a random selection will be chosen by the researchers to prevent selection bias. The selected coded names will be transferred back to the therapists, who will then provide the contact information of these patients to the researcher and will inform the patients that an assessor will approach them. The assessor will call the patient and answer any questions the patient may have about the study. Every participant has a two-week period to consider participation. If the patient is still willing to participate after this period, an assessor will make an appointment for an intake to confirm that the patient fulfills all of the study’s inclusion criteria. The intake will take approximately 1,5 hours. Patients that are included in the study will be randomly allocated to the treatment condition (MERIT) or treatment as usual (TAU). The randomization is conducted separately for each center and procedures will start directly after the first participants are included. Randomization will be done by a party independent from the trial research team. Results of the randomization process will be passed on to the onsite therapists. Block randomization will be used to ensure that the number of patients will be balanced over condition. Assessors will be blind to the subject’s condition. Rater blinding will be verified after the assessment. For therapists, it is of course not possible to be blind for condition. After assignment to intervention or control condition, the assessor plans a second appointment with the patient for baseline assessment. A research assistant, blind to treatment or control condition, who is not involved in the patient's treatment, will carry out this assessment. This assessment will take approximately 1.5 hours. Thereafter, the individual treatment will start. Onsite clinical psychologists that have partaken in the three-day MERIT training will provide the intervention. Participants in the control condition will receive treatment as usual (TAU). After forty sessions of treatment or forty weeks in the control condition, the assessment will be repeated (post-treatment). Follow-up assessment will take place six months after completion of the intervention.

### Materials

A summary the design of the study and all materials is provided in the table below (Table 1).

**Table 1:** Study design

**Instrument:**	**Selection**	**Intake**	**Assessment to condition**	**Baseline measures**	**Intervention**	**Post- treatment**	**Follow-up**
Four questions for the therapist	X						
MINI-PLUS		X					
IPII		X				X	X
MAS- NL		X				X	X
PANSS		X				X	X
Randomization			X				
Faux Pas test				X		X	X
Empathic accuracy test				X		X	X
IRI				X		X	X
QIDS-SR				X		X	X
BCIS				X		X	X
ISMI				X		X	X
MANSA				X		X	X
Time-use				X		X	X
WorQ				X		X	X
NLV				X		X	X
Trailmaking A&B				X		X	X
Digit symbol				X		X	X
Tic-P				X		X	X
EQ5D				X		X	X
MERIT therapy or control					X		

#### Metacognitive reflection and insight therapy (MERIT)

The experimental condition will receive the Metacognitive Reflection & Insight Therapy [based on 26]. This manual-based individual psychotherapy aims to stimulate the four elements of metacognition. The treatment protocol is not a step-by-step program, but is target-driven. The therapist tries to elicit a narrative; a personal story of the patient. In this personal narrative, the therapist looks for targets; signs of metacognition. Is the patient aware of his thoughts? Can he reflect on those thoughts and on the thoughts of others? The levels of MAS-A are used here to classify the different levels of metacognitive functioning. The therapist adjusts his interventions according to the level of metacognition of the patient and stimulates the patient to perform ever more complex metacognitive tasks. The therapy will consist of 40 times 45 minutes of individual therapy sessions. The treatment protocol has been translated into Dutch by the research team. Every participating therapist will follow a three-day training in the therapy provided by PL, the first author of the therapy manual. The most important aspects of the therapy are summarized in the Therapist Metacognitive Adherence Scale (T-MAS, see Table 2). The eight core items of the T-MAS are explained in detail in the protocol and the therapists will practice these elements in the training. The therapist has to give him/herself a score on a ten-point scale on the items of the T-MAS after every therapy session. In this way the therapist can monitor his/her own adherence to the aspects of the therapy. Transcripts of sessions will be selected at random and will be scored by independent assessors with the T-MAS, to monitor if the therapist follows the elements of the therapy correctly. Every other week each therapist will receive supervision by the developer of the therapy, PL, using Skype. In these sessions the adherence to the therapy elements and the T-MAS scores will be discussed and the therapists receive supervision on how they conduct the therapy.

**Table 2:** The therapist metacognitive adherence scale

Openness to the patient’s agenda at the session outset and throughout the session.	1	2	3	4	5
Offer of the therapist’s thoughts/perceptions regarding the patient’s behavior in the session.	1	2	3	4	5
Details of a narrative episode are elicited.	1	2	3	4	5
A psychological problem or dilemma is framed as something to be discussed.	1	2	3	4	5
Reflection on the interpersonal processes during the session is elicited.	1	2	3	4	5
Reflection on progress/course of the session is elicited at various times during the session or at session’s end.	1	2	3	4	5
The patient is stimulated to engage in metacognitive acts with interventions that are appropriate to patient’s capacity for self-reflectivity and/or awareness of the mind of the other.	1	2	3	4	5
The patient is stimulated to engage in metacognitive acts with interventions that are appropriate to patients’ capacity for metacognitive mastery.	1	2	3	4	5

**Key:** 1. Absent; 2. Intermittent moments in which basic competency is present; 3. Fully adequate or competent throughout; 4. Fully adequate with some periods of exceptional performance; 5. Consistently exceptional performance.

#### Screening

On-site therapists will screen all patients in their caseload that meet the inclusion criteria; they will answer four screening questions concerning each of these patients’ metacognitive abilities:

– To what extent is the patient able to think about his/her own thoughts?

– To what extent is the patient able to explain his/her thoughts and feelings in an adequate manner?

– To what extent is the patient able to explain the thoughts and feelings of others in an adequate manner?

– To what extent is the patient able to use his/her understanding of his/her own thoughts and feelings and the thoughts and feelings of others to react adequately during stressful events?

The therapists will answer these questions on a 10-point scale, 10 meaning excellent, 0 very poor. They select all the patients that score below 6 on two or more of the four questions, who will be approached for participation.

#### Intake

**Diagnosis***M.I.N.I Plus Interview*[37]. This structured interview is used to confirm a diagnosis of schizophrenia or schizoaffective disorder. It is designed to detect Axis I diagnoses of the DSM IV-TR. The interview is divided into 26 sections; each section concerns a diagnostic category. At the end of every category the interviewer can determine whether the patient fulfills the diagnostic criteria for the diagnosis of the particular section.

#### 

**Metacognition***Indiana Psychiatric Illness Interview*[38]. This interview, which consists of five open questions, was developed with the goal of eliciting the life story and illness history of the patient. The MAS-A can be scored on the resulting transcript.

*Metacognition Assessment Scale*[10,11]. The MAS-A consists of four scales. The cutoff points for each scale: Selfreflectivity: below 5.5, Understanding the other’s mind: below 4.5, Decentration: below 2, Mastery: below 4.5. When the patient gets a score below cut off on three or more scales, metacognitive deficits are prominent and the patient meets the inclusion criteria.

#### 

**Symptoms***Positive and Negative Syndrome Scale*[39]. This structured interview consists of thirty items, each ranging from one to seven. The items fall into three subscales: positive symptoms, negative symptoms and general symptoms.

#### Assessment

**Metacognition***Beck Cognitive Insight Scale (BCIS)*[40]. This self-report questionnaire evaluates the reflectiveness and overconfidence in the interpretation of experiences of the patient. It consists of fifteen items, six items represent the self-certainty subscale, nine items represent the self-reflectiveness subscale.

*Faux Pas Task*[41]. This task consists of ten stories, describing interpersonal, everyday situations. Some of these stories contain a ‘faux pas’: a speaker in the story says something without considering if it is something that the listener might not want to hear. The patient has to detect these mistakes.

*Empathic Accuracy Task (EAT)*[42]. This task consists of ten videos of people who tell a personal story. The participant has to rate the mood of the person in the video continuously on a 9 point scale.

*Interpersonal Reactivity Index* (IRI) [43]. This questionnaire exists of 28 statements. The participant has to indicate whether the statement applies to him/her on a six point scale.

#### 

**Depression***The Quick Inventory of Depressive Symptomatology Self Report (QIDS-SR)*[44]. This self-report questionnaire consist of sixteen items, measuring depressive symptoms according to the DSM-IV in the week before assessment.

#### 

**Stigma***The Internalized Stigma of Mental Illness Scale (ISMI)*[45]. This is a self-rating questionnaire with 29 questions designed to measure the subjective experience of stigma. It consists of five subscales: Stereotype Endorsement, Perceived Discrimination, Social Withdrawal, Stigma Resistance and Alienation.

#### 

**Quality of life***Self-rating Manchester Short Assessment of Quality of Life (MANSA)*[46]. This questionnaire consists of four objective questions and twelve subjective questions. These subjective questions assess satisfaction with life as a whole, job, financial situation, number and quality of friendships, leisure activities, accommodation, personal safety, people that the individual lives with, sex life, relationship with family, physical health and mental health. It is a self-report, 16 Likert-scale item measure.

#### 

**Social functioning***Time Use*[47]. This structured interview investigates the way in which the patient spends his/her time and makes a detailed account of his/hers: employment, education and training, voluntary work, leisure activities, hobbies, child care, housework and chores.

#### 

**Work readiness***Work Readiness Questionnaire (WoRQ)*[48]. This questionnaire is filled in by the therapist of the client and has seven items designed to capture the patient's readiness to work as reflected by current capacity to initiate and maintain a useful activity. The items are rated to provide graded measurements, i.e., “strongly agree”, “agree”, “disagree” or “strongly disagree”. Next to these seven items it contains a final dichotomous work readiness judgment.

#### 

**Neurocognition***Nederlandse Leestest voor Volwassenen (NLV)*[49]. The NLV tests the pronunciation of irregularly spelled words and is used to determine premorbid intelligence.

*Trailmaking test A&B (TMT)*[50]. The TMT provides information on visual search, scanning, mental flexibility speed of processing and executive functions. It is part of the Halstead–Reitan Battery [50]. The TMT consists of two parts. Part A requires an individual to draw lines sequentially connecting 25 encircled numbers distributed on a sheet of paper. Task requirements are similar for Part B except the person must alternate between numbers and letters (e.g., 1, A, 2, B, 3, C, etc.). The score on each part represents the amount of time required to complete the task [51].

*Digit Symbol Test* (part of the Wechsler Adult Intelligence Scale [52]). This test evaluates the recognition and recoding of visual information. The test consists of several rows of paired boxes with a digit in the top box and an empty space in the box below. At the top of the page is shown which symbols are paired to the digits. The participant has to fill in as many symbols in the empty boxes within 90 seconds.

#### 

**Cost-effectiveness***EuroQol (EQ-5D)*[53]. This questionnaire contains five scales: self care, mobility, usual activities, pain/discomfort and anxiety/depression. The participant has to rate his/her health as it is on the day of the interview, using five levels: “no problem” “slight problems” “moderate problems”, “severe problems”, “unable”/”extreme problems” [54].

*TiC-P*[55]. This is a questionnaire. The first part consists of medical resource items, including: contacts within the mental healthcare sector, self-help groups, contacts with general healthcare providers, and the use of medication. The patient has to indicate whether he/she makes use of these items and if yes, how many times. Depending on the relevance for the target population, the questionnaire allows adding or leaving out specific items of resource utilization. Part two consist of the Short Form-Health and Labour Questionnaire (SF-HLQ) an instrument that collects data on productivity losses due to health problems. This part measures absence from work and reduced.

## Statistical analysis

### Descriptive statistics

Descriptive statistics will be provided for all normally distributed variables in the form of mean scores (and standard deviations) before and after the intervention on each of the assessment instruments. Descriptive statistics of variables that are not normally distributed will be represented as median scores and ranges.

### Analysis

Differences in scores on every dependent variable are measured before and after the intervention and six months after the end of intervention. The significance of possible differences will be tested using logistic multileveling modeling [56] with the subject and assessment time as levels and condition as independent variable. A model will be constructed for every dependent variable using the program MlwiN [57]. Dummy variables will be constructed for every level and the statistical significance of the regression-effects will be tested using the T-test. The dummy-variables and their interactions will be added to the model as fixed effects.

## Discussion

With this multicenter randomized controlled trial, we intend to investigate the effectiveness of a manual-based psychotherapy aimed at improving metacognition and daily life functioning of patients with schizophrenia. Metacognition is a complex, multi-dimensional concept, involving four main aspects: self reflection, understanding the mind of the other, decentration and mastery. It not only refers to the ability to detect and consider discrete psychological phenomena (for example: recognizing a thought), but also to the ability to integrate these phenomena into an integrated, complex representation of self and others [12]. MERIT tries to enhance these synthetic metacognitive processes through constant reflection in the interpersonal relationship. In this way, the patient learns to reflect on him/herself in ever more complex ways. We hope this will result in structural changes in metacognitive processes in patients with schizophrenia, and hope this in turn will lead to structural, lasting improvements of daily life functioning of the patient. This design brings along some methodological difficulties, especially the risk of drop-out over time. We hope to account for this by including 20% extra patients in the trial to ensure statistical power will be maintained despite patient attrition.

Most interventions for people with schizophrenia in the Netherlands target first-episode patients within five years after the first psychotic episode. Care for chronic patients usually focuses on support and structure instead of recovery. MERIT can be used with patients who are chronically mentally ill, and case studies show promising results. If proven effective, this therapy might be a useful addition to the care for chronic schizophrenia patients.

Furthermore, some patients benefit less from usual interventions such as psycho-education and cognitive behavioral therapy. It could be hypothesized that these patients are not able to form complex representations of self and others, which makes it difficult to engage in therapy. Improving metacognition might prove a necessary step for these patients to benefit from other interventions and enhance daily functioning.

## Competing interests

The authors declare that they have no competing interests.

## Authors’ contributions

SJ, MP and RD conceived the study and designed the study with advice from PL, LW, MG and AA. PL designed the treatment protocol. SJ, MP and RD translated the protocol into Dutch. All authors have read and approved the final manuscript.

## Pre-publication history

The pre-publication history for this paper can be accessed here:

http://www.biomedcentral.com/1471-244X/14/27/prepub
